# Rosemary Extract and Essential Oil as Drink Ingredients: An Evaluation of Their Chemical Composition, Genotoxicity, Antimicrobial, Antiviral, and Antioxidant Properties

**DOI:** 10.3390/foods10123143

**Published:** 2021-12-18

**Authors:** Spyridoula D. Christopoulou, Chrysa Androutsopoulou, Panagiotis Hahalis, Chrysoula Kotsalou, Apostolos Vantarakis, Fotini N. Lamari

**Affiliations:** 1 Laboratory of Pharmacognosy & Chemistry of Natural Products, Department of Pharmacy, University of Patras, 26504 Patras, Greece; chem2832@upnet.gr; 2 Department of Public Health, Faculty of Medicine, University of Patras, 26504 Patras, Greece; chrysandr@gmail.com (C.A.); chrysoulakotsalou@gmail.com (C.K.); avanta@upatras.gr (A.V.); 3 Tentoura Castro-G.P. Hahalis Distillery, 26225 Patras, Greece; hahalis@tentoura.gr

**Keywords:** rosemary, essential oil, extract, antimicrobial, antiviral, acute toxicity, antioxidant, chemical characterization, eucalyptol

## Abstract

*Rosmarinus officinalis* L. (rosemary) is in high demand in the food and drink industries due to its distinct organoleptic properties. With the aim of evaluating the rosemary leaves as drink ingredients, both the essential oil and alcoholic (38%, *v*/*v*) extract were studied in terms of chemical composition, genotoxicity, antimicrobial, antiviral, and antioxidant properties. GC–MS analysis showed that the main volatile compounds in the essential oil were eucalyptol (40.1%), camphor (12.4%), and α-pinene (12.9%). LC–MS analysis revealed gallocatechin and rosmarinic acid as the main extract ingredients. Both the essential oil and the extract were not genotoxic (Ames test) against TA98 and TA100 at the dilutions of 5% and 90%, respectively; those dilutions were selected as the maximum possible ones in the drink industry. Their activity was investigated against *Escherichia coli*, *Salmonella enterica* serovar Typhimurium, *Staphylococcus aureus*, *Aspergillus niger*, and Adenovirus 35. Both were effective against Adenovirus and *A. niger*, even the essential oil at 5% (*v*/*v*). The extract at dilutions of 25–90% had more pronounced activity against tested bacteria than the essential oil at the dilutions of 5–100%; the essential oil at the dilution of 5% inhibited *S. aureus* growth. The antioxidant activity was evaluated by the 2,2-diphenyl-1-picrylhydrazyl radical scavenging assay, the 2,2-azino-bis-3-ethylbenzothiazoline-6-sulfonic acid decolorization assay, and the ferric reducing antioxidant power assay. Both exhibited good antioxidant activity, but rosemary essential oil was far more effective than the extract. Our results demonstrate that rosemary essential oil and extract are safe and have beneficial biological properties. Therefore, they could serve as health-promoting ingredients in the drink industry.

## 1. Introduction

*Rosmarinus officinalis* L. (Lamiaceae family) is widely cultivated for its leaves. It has been used as a stimulant, as an analgesic, and against inflammatory diseases, physical and mental fatigue in traditional medicine [1]. The pharmacologically validated medicinal properties of rosemary include antibacterial and antioxidant [1,2], antiviral [3], anti-inflammatory [4], antifungal [5], and antiproliferative effects toward cancer cells, absence of genotoxicity, and the ability to prolong thrombin time [6]. The antidepressant properties have been studied in various models [7,8]; we have earlier shown that rosemary infusion has antidepressant/anxiolytic-like and anticholinesterase effects [9]. In 2018, an intervention study in healthy adults showed that rosemary water consumption had beneficial cognition effects, which agreed with those previously discovered for inhaling the scent of rosemary essential oil [10].

The Food and Drug Administration (FDA, Silver Spring, MD, USA) has listed many essential oils as “substances generally recognized as safe” for consumption in foods and beverages [11]. European Food Safety Authority (EFSA, Parma, Italy) has also authorized rosemary extracts as a food additive (E 392) and the range of margins of safety was 100–2000 and 200–3000 mg carnosol plus carnosic acid for children and adults, respectively [12]. Due to the high demand for it as a food additive and a food/drink ingredient, the rosemary extracts market was estimated at USD 215 Mn in 2019 and is anticipated to grow at an annual rate of 3.7 percent between 2020 and 2025 [13]. 

Apart from its distinct favorable organoleptic properties, many studies have demonstrated beneficial antimicrobial and antioxidant properties of the rosemary essential oils and extracts justifying their use as natural preservatives [14]. Specifically, their addition can effectively inhibit the oxidative processes in the processing and storage of meat products such as salami and fresh chicken and maintain/improve the product’s organoleptic properties [15]. However, their value as natural ingredients in the drink industry has not been thoroughly investigated, although there is a trend in producing bitter alcoholic beverages from aromatic and medicinal plants [16]; in those, their content can be high.

Antibacterial and antioxidant activities of the essential oil stem from certain volatile categories, i.e., monoterpenes ketones, hydrocarbons, and oxides [2,17]; 1,8-cineole, camphor, and α-pinene are mainly responsible for that bioactivity [17,18,19]. With regard to the rosemary extracts, antimicrobial and antioxidant activities are attributed to the phenolic acids, flavonoids, and terpenoids [20,21], and particularly to carnosic acid and rosmarinic acid [22,23]. Many studies have shown the great variability of chemical compositions among different rosemary genotypes, environments, cultivation conditions, harvest times, and extraction conditions [24], revealing the need for thorough extract and essential oil characterization and thereafter standardization.

The aim of this study was the evaluation of rosemary essential oil and extract as ingredients in the drink industry. Thus, we proceeded to the full chemical characterization of rosemary essential oil and ethanolic extract and the investigation of their efficacy against bacteria (*E. coli*, *S. aureus*, *S. typhimurium*), fungus (*A. niger*), virus (Adenovirus 35) in different dilutions relevant to their potential use in final alcoholic beverages. 2,2-Diphenyl-1-picrylhydrazyl (DPPH) radical scavenging activity, 2,2-azino-bis-3-ethylbenzothiazoline-6-sulfonic acid (ABTS) decolorization, and ferric reducing antioxidant power (FRAP) assays were used to evaluate their antioxidant capacity. Finally, their toxicity was determined with the AMES test.

## 2. Materials and Methods

### 2.1. Essential Oils and Extracts

The essential oil and the herbal extract were produced by the distillery “Tentoura Castro-G.P. Hahalis” located in Patras, Greece. *Rosmarinus officinalis* L. was cultivated in Patras and was collected in July–August 2019. In an experimental 10 L distillery, the essential oil was isolated using water-steam distillation. Specifically, 0.6 kg of fresh rosemary leaves were distilled for 3–4 h in 8 L of water. Maceration was used to create the herbal extracts. Glass and stainless-steel containers were used for extraction. Rosemary leaves were dried for 8 days before being extracted for 25 days in 40 L of a solvent containing 38 percent *v*/*v* ethanol at a temperature of 20–25 °C.

### 2.2. Gas Chromatography–Mass Spectrometry

An Agilent Technologies 6890N GC instrument equipped with a 5975B mass selective detector (MSD) was used in the electron impact (EI) mode of 70 eV at the Central Instrumental Analysis Laboratory of the University. The carrier gas was helium, and the capillary column was HP-5MS (30 m × 0.25 mm, 0.25 μm). GC/MSD Chemstation (Agilent Technologies, Inc., Santa Clara, CA, USA) and MestreNova v.6.0.2-5475 were used to analyze the data (Mestrelab Research S.L., Santiago de Compostela, Spain). 

The initial GC oven was 50 °C for 2 min, which was then ramped at 2 °C/min to 80 °C for 1 min, at 8 °C/min to 100 °C for 1 min, at 10 °C/to 200 °C for 1 min. Finally, the oven warmed up to 300 °C at a rate of 10 °C/min and then kept at 300 °C for 5 min. The carrier gas was at a rate of 1.0 mL/min, in a splitless mode, and the mass range was *m*/*z* 40–1000.

The sample was diluted (1:40) in ethyl acetate, and the injection volume was 1 μL. n-Octane (98% purity) was utilized as an internal standard at a final concentration of 0.3 mg/mL. Alkanes (C8–C24) were examined under the same conditions and utilized as reference points for computing retention indices using the Van den Dool and Kratz equation [25]. By comparing the experimental retention indices (AI_cal_) and MS spectra to commercial databases [26,27] and the literature, the chemical components were identified. The standard compounds α-pinene (>99%), eucalyptol (>99%), linalool (>99%), and camphor (>99%) were purchased from Sigma-Aldrich (Steinheim, Germany) and were used for the verification of the identification. The results were expressed as a percentage of the ratio of each compound peak area to that of the internal standard using the program WSEARCH32 (Ver. 16/2005).

### 2.3. Liquid Chromatography–Mass Spectrometry

In this study, a single quadrupole LC/MS system, the LC/MSD1260 Infinity II (Agilent Technologies, Inc.), was used. The mass range of this system was *m*/*z* 100–1000, and it was equipped with an ESI ion source. Nitrogen was used as an ionization gas. A Poroshell 120 EC 18 column (4.6 × 100 mm, 2.7 μm) was used for separation (Agilent Technologies, Inc.). The following were the LC conditions: solvent A (0.1% formic acid) and solvent B (acetonitrile with 0.1% formic acid) and the gradient elution method was: 0–5 min 4% B; 5–15 min 4–15% B; 15–18 min 15% B; 18–23 min 15–20% B; 23–33 min 20% B; 33–53 min 20–58% B; 53–68 min 58% B; 68–80 min 58–95% B; 80–85 min 95% B; 85–89 min 95–4% B; 89–93 min 4% B. The injection volume was 20 μL, and the flow rate was 0.5 mL/min. Dilution in 1% formic acid to a final concentration of 2 mg raw plant material/mL was used to prepare samples for UPLC–ESI–MS.

The standards that were used for identification and/or quantification were rutin (HPLC > 99%) from Extrasynthese (Genay, France), quercetin 3-O-glucoside (HPLC > 98%) from Extrasynthese, kaempferol (HPLC > 96%) from Sigma Aldrich (Steinheim, Germany), rosmarinic acid (HPLC > 98%) from Extrasynthese, and carnosic acid (HPLC > 98%) from Extrasynthese. The rest of the compounds were identified by comparing their elution order and their mass spectra to those in the literature. The quantification of the flavonoid compounds was based on the rutin standard curve (3.125–100.000 μg/mL, y = 29,361x + 425,743, R^2^ = 0.9632), of the phenolic acids on the rosmarinic acid curve (2.50–100.00 μg/mL, y = 99,751x + 307,188, R^2^ = 0.9978), and of the diterpenes on the carnosic acid (1.00–25.00 μg/mL, y = 380,826x + 309,232, R^2^ = 0.9943).

### 2.4. Antioxidant Activity

The antioxidant activity was evaluated by the DPPH (1,1 diphenyl-2-picrylhydrazyl) radical method, the Ferric reducing antioxidant power (FRAP) assay and the ABTS [(2, 2′-azinobis-(3-ethylbenzothiazoline-6-sulfonate)] assay. Sample blanks (containing only the sample in the appropriate final volume) are used for every assay and their response was subtracted from that of the samples.

#### 2.4.1. DPPH Radical Scavenging Assay

The scavenging of the DPPH radical by the samples was determined with a slight modification of previous protocols [28]; briefly, 5 μL of the test materials (standard or sample) were mixed with 195 μL of 0.1 mM methanolic DPPH solution. Butylhydroxytoluene (BHT) was used as a standard. After an incubation period of 30 min at 25 °C in the dark, the absorbance at 540 nm was recorded as A_sample_. The radical scavenging activity was expressed as percentage inhibition of DPPH and was calculated according to the formula IC (%) = [(A_o_ − A_t_)/A_o_] × 100, where A_o_ and A_t_ are the absorbance values of the blank sample and the test sample, respectively. The radical scavenging activity of the samples was expressed as mg BHT/mL and it was based on the BHT calibration curve (0.025–40 mg/mL, y = 31.605x + 44.512, R^2^ = 0.9924).

#### 2.4.2. FRAP Assay

Based on a previous method [29], the samples’ ability to reduce ferric iron (Fe^3+^) was determined. Before each experiment, the FRAP reagent was freshly prepared and contained 300 mM of acetate buffer (pH = 3.6), 10 mM 2,4,6-tri(2-pyridyl)-1,3,5-triazine (TPTZ) in 40 mM HCl, and 20 mM FeCl_3_×6H_2_O in pure water in a ratio of 5:1:1. Solutions of FeSO_4_×7H_2_O were used as standards. 60 μL of test materials (standards/plant essential oil and extracts/blank) were mixed with 55 μL 300 mM of acetate buffer (pH = 3.6) and 80 μL of freshly FRAP reagent and incubated for 5 min. Absorbance was determined at 594 nm. The FRAP values were expressed as mg FeSO_4_×7H_2_O/mL of sample, and it was based on the FeSO_4_×7H_2_O standard curve (0.014–0.278 mg/mL, y = 2.9543x + 0.0353, R^2^ = 0.9917). 

#### 2.4.3. ABTS Decolorization Assay

The ABTS test was performed using the method Tzima et al. [28], with minimal changes. Reagent stock solution was prepared by mixing 88 μL of 2.45 mM K_2_S_2_O_3_ in water with 7 mM of a methanolic ABTS solution to a final volume of 5 mL. For 12–16 h, the freshly made reagent solution was stored at room temperature in the dark. The solution was then diluted in ethanol (approximately 1:30, *v*/*v*) to an absorbance of 0.700 at 754 nm before the experiment. Solutions of Trolox were used as standards. Then, 5 μL of diluted samples/standards/blank and 245 μL of ABTS reagent were mixed and incubated for 5 min in the dark. The absorbance was measured at 754 nm. ABTS radical scavenging activity of the samples was expressed as scavenging activity (%) = [(A_sample_ − A_control_)/A_control_] × 100, where A_control_ is the absorbance of the blank control (ABTS solution without test sample), and A_sample_ is the absorbance of the test sample. The ABTS values were expressed as mg Trolox/mL and it was based on Trolox calibration curve (0.025–5 mg/mL, y = 2.9543x + 0.0353, R^2^ = 0.9917).

### 2.5. Mutagenicity Assay

The mutagenic potential was determined with the Ames test [30,31]. The kit Ames *Salmonella*/mutagenicity assay was provided by EBPI (Mississauga, ON, Canada), and the experimental procedure was described by Androutsopoulou et al. [32]. In brief, *Salmonella* strains TA98 and TA100 were cultured in growth media containing Express Reagent V. Dilutions of rosemary essential oil up to 5% in DMSO and of the extracts up to 90% in distilled water were used. The determination of toxicity was colorimetric at 600 nm. All experiments were performed in triplicate.

### 2.6. Antibacterial and Antifungal Assay

Antibacterial activity was evaluated using the agar dilution technique against a group of bacterial strains, as recommended by the National Committee for Clinical Laboratory Standards [33]. Experiments were performed on *Escherichia coli* NCTC 9001, *Salmonella typhimurium* NCTC 12023, and *Staphylococcus aureus* NCTC 6571 (Sigma-Aldrich, St. Louis, MI, USA), as described by Androutsopoulou et al. [32]. Antifungal activity against *Aspergillus niger* (Sigma-Aldrich) was also performed by daily monitoring of the diameter of each colony as described by Androutsopoulou et al. [32]. All experiments were performed in triplicate at each concentration.

### 2.7. Antiviral Assay

The antiviral activity of the samples against human Adenovirus serotype 35 (AdV) at concentrations that were not cytopathic in A549 cells (Life Science Chemilab, Athens, Greece), P +92 generation, was determined as previously described [32]. In brief, AdV in serial dilutions (four replications of each dilution) or the samples in different concentrations were propagated in A549 cells and the effect on cell viability was recorded. For the antiviral activity, the cytotoxicity was observed by electron microscope, according to Saderi et al. (2011) [34]; the sample concentration that entirely suspends AdV35 cytopathic effect is recorded as efficient concentration, compared with virus control. All experiments were performed twice.

### 2.8. Data Analysis

All data are expressed as mean ± standard deviation of all replicates. The data were organized, analyzed, and visualized with Microsoft^®^ Excel. If a difference is reported, it means that it is statistically significant at *p* < 0.05.

## 3. Results and Discussion

### 3.1. Chemical Analysis

In the essential oil of rosemary, 20 compounds were identified, representing 99.81% of the total oil (Table 1, Supplementary Materials Figure S1). The main components of the essential oil were eucalyptol (1,8-cineole) (40.10%), camphor (12.40%), α-pinene (12.94%), β-pinene (8.94%), and camphene (6.38%).

**Figure 1:**
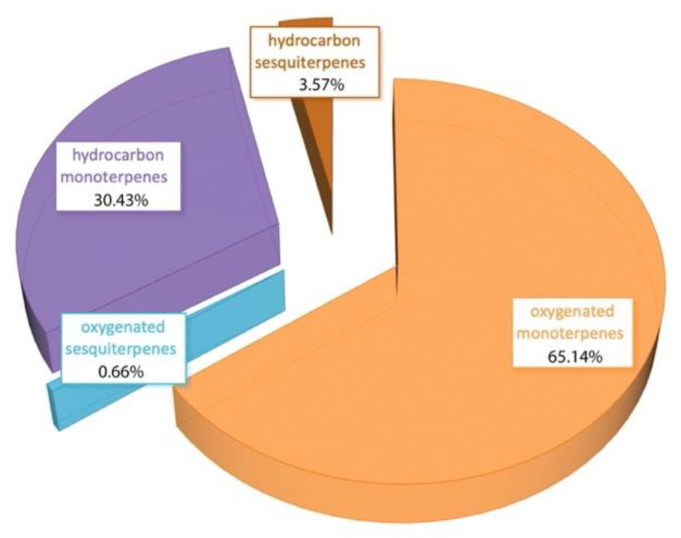
Percentage of each category of volatile compounds in rosemary essential oil.

The great majority of essential oil compounds were monoterpenes (95.57%), while the sesquiterpenes amounted only to 4.24%, as can be seen in Figure 1. The results of our analysis are in line with previous studies of rosemary in Greece and abroad. Studies show great variability of the relative abundance of 1,8-cineole, camphor, and a-pinene suggesting the existence of 1,8-cineole and camphor-chemotypes, and two intermediate types, i.e., camphor/1,8-cineole/borneol type and 1,8-cineole/camphor type [35]. The sample analyzed herein clearly belongs to the 1,8-cineole chemotype. 

**Figure 2:**
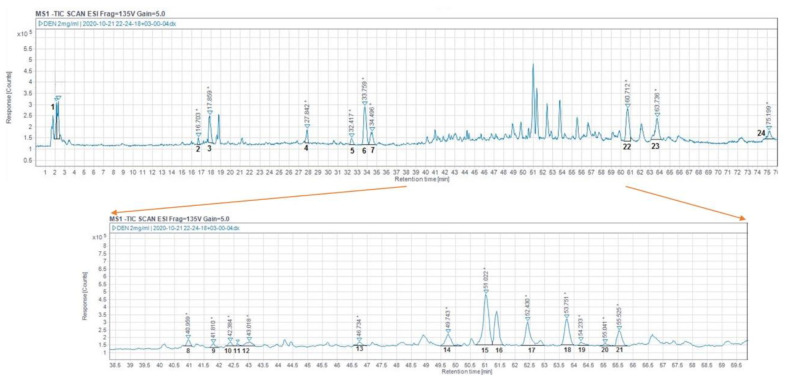
Total ion chromatogram for LC–MS analysis in negative ionization mode of rosemary leaves extract. The whole chromatogram is shown in the upper panel and a zoomed-in section is presented in the lower panel. Peaks are numbered as in Table 2.

The rosemary extract analysis showed 24 ingredients (Table 2, Figure 2). Compounds were identified by comparison of their retention time and mass spectra with those of the standards and the literature data; those compounds were terpenoids (peaks 14–21, 23, 24), flavonoids (peaks 3–5, 7–11, 13), and phenolic acids (peaks 1, 2, 6, 12), whereas peak 22 could not be identified. The flavonoids were the predominant category of compounds (54.4%) that were quantified, followed by the phenolic acids (26.7%) and the diterpenes (17.1%); triterpenes were not detected. In this study, nine flavonoids (compounds 3–5, 7–11 and 13) were identified in accordance with earlier studies [23,36,37,38]. In particular, peak 9 (luteolin-3-O-acetyl-β-glucuronide) has also been reported in *R. οfficinalis* from Serbia [37], peak 10 (luteolin) in *R. οfficinalis* from Spain [38], and peak 12 in an ethanolic extract from Mexico [29]. Finally, in this study, four phenolic acids were identified in line with Queralt et al. [39] and Perez-Mendoza et al. [29]. 

The main ingredients of the herbal extract were gallocatechin (26.4%), rosmarinic acid (18.0%), and luteolin-3-*O*-acetyl-*O*-glucuronide (14.2%). Carnosol was detected but could not be quantified, carnosic acid was not detected, and only 12-*O*-methyl carnosic acid was in low levels (0.5%). High seasonal variability in the content of carnosic acid was earlier shown by Fumiere Lemos et al. [24], which might be one reason for our findings, along with genotype and extraction parameters.

### 3.2. Antioxidant Activity

The evaluation of the antioxidant capacity with three different assays demonstrated that the essential oil had significantly (*p* < 0.001) higher antioxidant activity in all cases (Table 3).

In a previous comparative study [24], rosemary essential oil was not an efficient antioxidant in the DPPH assay, whereas the extract was a good one, but the composition in both essential oil and extract was different from that described herein especially in the content of 1,8-cineole and carnosic acid, showing the need for complex mixture characterization every time a biological property is studied. According to Bereta et al. [40], the difference in radical scavenging activity among rosemary essential oils is primarily due to the varying amounts of major compounds in the essential oils. According to Insawang et al. [41], 1,8-cineole, when compared with other monoterpenes, is the most effective at reducing reactive oxygen species. Moreover, Goze et al. [42] demonstrated that these terpenes have similar antioxidant properties to those obtained from phenolic mixtures. Rosemary essential oil with the same composition was an effective antioxidant in vitro and in vivo [43]; the per os administration of the essential oil to rats at a 10 mg/kg body weight for 7 days before the administration of CCl_4_ attenuated the hepatotoxicity markers, preserved renal function and the liver antioxidant status. Similar beneficial results of the rosemary essential oil were noted against hexavalent chromium toxicity [44].

Concerning the extracts, Kontogianni et al. have demonstrated that the antioxidant activities of commercial extracts depend on the concentration of phenolic abietane diterpenes, such as carnosic acid and its derivative carnosol and rosmanol isomers [23]. In our extract analysis, the terpenoids were the most abundant compounds. Yeddes et al. [45] reported that bioclimatic and season conditions (especially temperature) can influence the concentrations of rosmarinic acid and carnosic acid and thus the antioxidant potential of rosemary plant extracts; the highest antiradical activity of rosemary leaf extracts was obtained in summer. The plant material used in this study was collected in summer as well, and it had a good antioxidant activity with the DPPH assay (IC50 value of 12.32 mg/mL). Earlier studies in male hamsters that followed a high-fat diet supplemented with rosemary ethanolic extract demonstrated that rosemary was an efficient antioxidant in vivo, alleviating high-fat-induced oxidative damage, as well as enhancing the antioxidant enzyme activity, gene, and protein expression level via regulating the Nrf2 pathway [46]. More importantly, when rosemary in the form of dry powder (500 mg) was administered to healthy human volunteers twice a day for a month, plasma acetylcholinesterase was decreased, plasma total antioxidant activity was increased, and protein carbonylation was decreased in comparison with the values before treatment and the placebo group [47].

Conclusively, both essential oil and extract had good in vitro antioxidant activity; although the essential oil had higher antioxidant activity, it is used in lower percentages than the extract; in the scenario of essential oil usage at 0.5% and extract of 90% they could still have antioxidant activity, which would be comparable. 

### 3.3. Antimutagenesis

The essential oil up to the concentration of 5% was able to inhibit mutations induced in TA98 and TA100 *Salmonella* strains as well as the extract did at concentrations up to 90% (Supplementary Materials Table S1). Therefore, they were both not genotoxic in the studied range of dilutions. Earlier, it was shown that rosemary essential oil was genotoxic in peripheral mononuclear cells in a dose-specific way [48]. In a study evaluating the frequency of micronuclei in different cell lines after exposure to the extract for 24 h, it was also shown that all tested concentrations did not stimulate DNA damage and thus were not mutagenic [49]. Razavi-Azarkhiavi et al. [50] also concluded that the constituents of the ethanol extract of rosemary prevented human lymphocyte oxidative DNA damage. Moreover, in a clinical study in 67 HIV patients receiving antiretroviral therapy and concomitant rosemary extracts, the administration of the extracts reduced the genomic instability in oral epithelial cells [51].

### 3.4. Antimicrobial and Antiviral Properties

**Figure 3:**
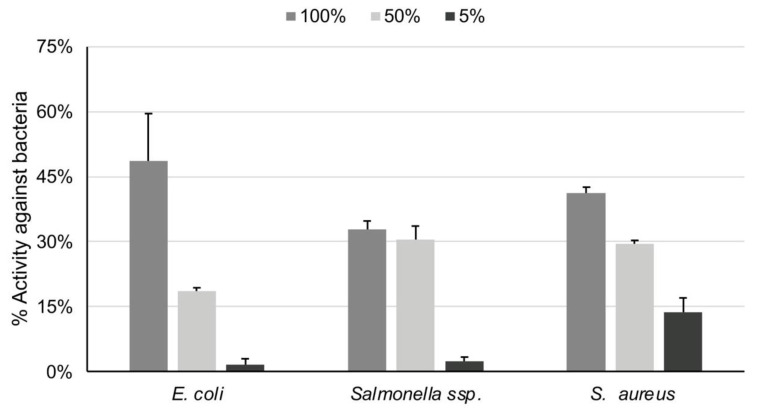
Antibacterial activity of rosemary essential oil against *E. coli*, *Salmonella* typhimurium, *S. aureus* in three different concentrations (5, 50 and 100%).

**Figure 4:**
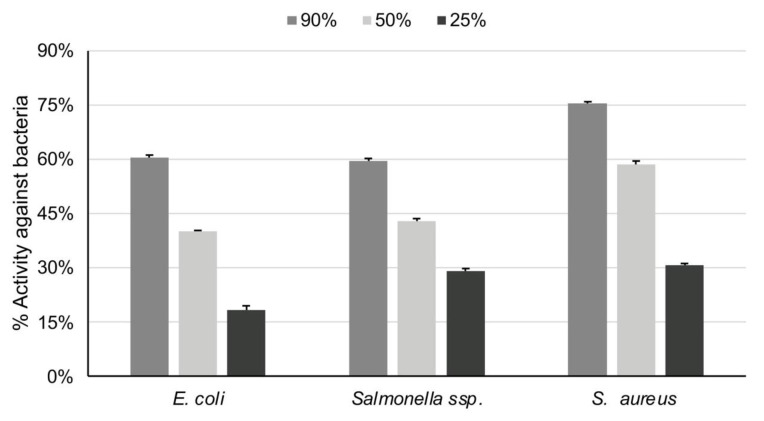
Antibacterial activity of rosemary extracts (90%, 50%, 25%) against *E. coli*, *Salmonella* typhimurium, and *S. aureus*.

The antibacterial activity of both the essential oil and the extract was dose dependent (Figure 3 and Figure 4). *Rosmarinus officinalis* essential oil revealed low-to-modest antibacterial activity against *E. coli*, in all trial concentrations; its ability in 100% concentration was 49%. The extract was more active (64% in the dilution of 90% and 55% in that of 50%) against *E. coli*. Regarding the antibacterial activity against *S. aureus*, the essential oil had a low activity even at the dilution of 5%, whereas the extract reached 74% in 90% concentration and 58% in 50%. Lastly, the essential oil also had low efficiency against *Salmonella typhimurium*; the extract on the other hand reached 59% in the concentration of 90%.

**Figure 5:**
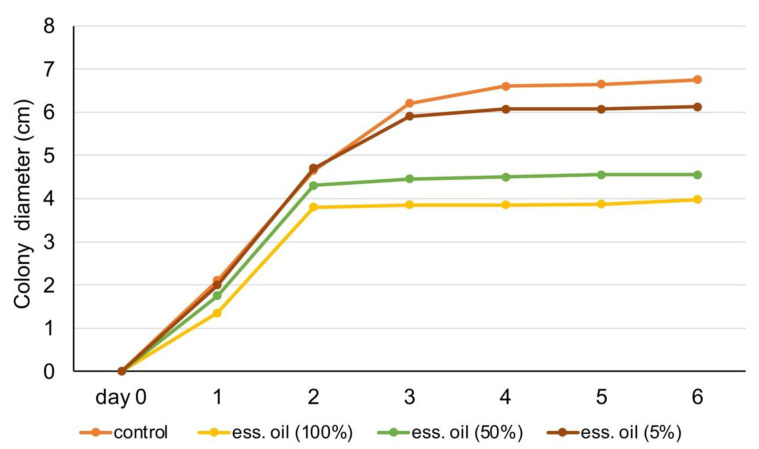
Antifungal activity of rosemary essential oil (100%, 50%, 5%) against *A. niger.*

**Figure 6:**
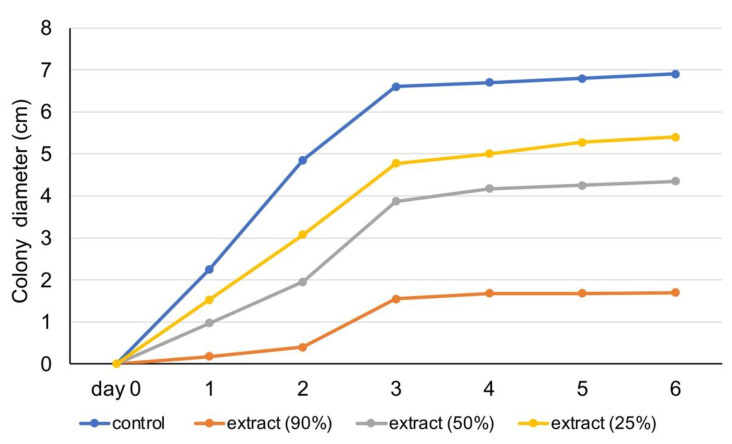
Antifungal activity of rosemary extract (90%, 50%, 25%) against *A. niger.*

In Figure 5 and Figure 6, experimental data show the fungal colony diameter (in cm) over one week. Results show that rosemary essential oil (100% and 50%) inhibited fungus colony growth. Specifically, by day 3, the diameter of colony development stabilized when treated with those essential oil dilutions, whereas in the control, it increased until it occupied the entire Petri dish, slowing its growth rate even more in the last days (Figure 5). Figure 6 shows the results of experiments on the rosemary extract. All three dilutions of the extract were efficient in limiting the growth of fungus (in comparison with control), with 90% presenting the highest antifungal activity. 

Cytotoxicity assay results are shown in Table 4 The essential oil in the concentration of 5% was not cytopathic, while the extract was not even at 100% concentration. The effects of essential oil (5%) and extract against Adenovirus at concentrations of 10^9^ PFU/mL to 10^4^ PFU/mL are shown in Table 5. The extract efficiently prevented the AdV35 cytopathic effect in all viral concentrations. In addition, the essential oil, despite its low concentration (5%), was effective against all Adenovirus concentrations.

Previous studies have demonstrated that rosemary essential oils have antimicrobial and antiviral activities [2,17,18,19,52,53,54,55]. Our results are in line with those studies and demonstrate, for the first time, the activity against Adenovirus 35. The essential oil studied herein with high 1,8-cineole content had a dose-dependent and modest antibacterial effect. Bajalan et al. [2] have demonstrated significant positive correlations of volatile oxides in rosemary essential oil and with the inhibition zone on *S. aureus*, and of ketones with that on *E. coli*. Moreover, in a previous study, 1,8-cineole was effective only against Gram-negative bacteria (*E. coli, K. pneumoniae*) inducing cell membrane disruption and a-pinene was against both Gram-positive (*S. aureus, E. faecalis*) and Gram-negative bacteria, whereas the effect of the rosemary essential oils was dependent on the composition [17]. Concerning the antifungal activity of the rosemary essential oil, our results confirm earlier studies [19,52]. Interestingly, in the study of Jianga et al. [19], the essential oil showed pronounced antifungal activity, compared with the pure compounds 1,8-cineole and α-pinene against the same fungus. With regard to the antiviral activity, we herein show the important anti-adenoviral activity of the essential oil. Accordingly, it has been earlier shown that rosemary 1,8-cineole chemotype significantly reduced the hepatitis A virus titers [53]. Due to the lipophilic nature of the volatiles in essential oils, they have the potential to infiltrate the lipid bilayer of the viral envelope [54]. Monoterpenes, the major compound class in rosemary oil, increase the fluidity and permeability of the cytoplasmic membrane, and disrupt the order of membrane-embedded proteins [55].

In our study, the rosemary extract was drastic against *E. coli*, *S. typhimurium*, *A. niger*, and Adenovirus. The rosemary extracts were potent against Gram-positive and Gram-negative bacteria in a study by Mahmood et al. [56]. In our experiment, rosemary extract displayed antifungal action against *A. niger* at concentrations ranging from 25 to 90%, which is consistent with the findings of Swari et al. [57], who demonstrated that rosemary ethanol extract had an inhibitory effect against *Candida albicans*. Rosemary extract had antiviral efficacy against HSV-1 and HSV-2, according to Al Mergin et al. [3]. Moreno et al. [20] proposed that the antibacterial efficiency of rosemary extracts was linked to their unique phenolic makeup, with antimicrobial action attributed mostly to carnosic acid and rosmarinic acid. The inhibitory effect of rosemary extract, according to Nieto et al. [1], is due to the activity of rosmarinic acid, rosmaridiphenol, carnosol, epirosmanol, carnosic acid, rosmanol, and isorosmanol. The primary components of rosemary extract in our investigation were rosmarinic acid and its derivatives. They interact with the cell membrane, altering the transport of electrons, causing changes in genetic material and nutrients, leakage of cellular components, and production changes in fatty acids [1].

## 4. Conclusions

This study provides, for the first time, an overall assessment of rosemary essential oil and extract in the concentrations that could be used in the drink industry in terms of safety, antioxidant, antimicrobial, and antiviral properties. Monoterpenes are the major compounds of rosemary essential oil, especially 1.8 cineole. The main components of the ethanolic rosemary extract belong to the class of flavonoids. The essential oil in dilutions of up to 5% and the extract in the range of 25–90% are not genotoxic, and confer antiviral, antifungal, and antioxidant properties; the extract also confers important antibacterial properties, whereas the essential oil is mainly effective against *S. aureus*. It can be concluded that those raw materials are safe for use in the drink industry and could impart important health benefits apart from their distinct sensory qualities.

## Figures and Tables

**Table 1:** List of volatile compounds in the essential oil of the rosemary leaves. Their % content is presented along with the experimental and literature retention indices.

Number	Volatile Compounds	AI_cal_	AΙ_lit_	% Peak Area/IS Area
1	Tricyclene	918	921	0.22 ± 0.00
2	α-Pinene ^st^	929	932	12.94 ± 0.49
3	Camphene	945	946	6.38 ± 0.12
4	β-Pinene	974	974	8.94 ± 0.19
5	Myrcene	991	988	1.37 ± 0.02
6	3-Carene	1008	1008	0.32 ± 0.00
7	p-Cymene	1022	1020	0.17 ± 0.00
8	Eucalyptol (1,8-cineole) ^st^	1032	1026	40.10 ± 0.65
9	Terpinolene	1085	1086	0.08 ± 0.00
10	Linalool ^st^	1092	1095	1.41 ± 0.05
11	Camphor ^st^	1151	1141	12.40 ± 0.27
12	Borneol	1170	1165	5.31 ± 0.21
13	Terpinen-4-ol	1179	1174	1.31 ± 0.08
14	α-terpineol	1195	1186	3.07 ± 0.15
15	Myrtenol	1195	1194	nq
16	Carvone	1249	1239	nq
17	Bornyl acetate	1288	1284	1.54 ± 0.06
18	E-Caryophyllene	1414	1417	3.45 ± 0.07
19	α-Humulene	1461	1452	0.13 ± 0.01
20	Caryophyllene oxide	1578	1582	0.66 ± 0.11
	number of components			20
	% total identified			99.81 ± 2.50

Notes: IS: internal standard, nq: not quantified, ^st^: the respective standard compound was used for the identification. Results are presented as mean ± standard deviation from triplicate analysis. AI_cal_: Experimental retention index on the HP-5MS column. AI_lit_: Literature retention indices on apolar column from Adams et al. (2012) [26].

**Table 2:** LC–MS identification and quantification of metabolites of the extract of rosemary leaves. Concentration is presented as mean ± standard deviation derived from triplicate analysis.

Peak No.	Rt (min)	Negative Ionization (*m*/*z*)	Positive Ionization (*m*/*z*)	M.W.	Molecular Formula	Tentative Identification	Concentration (μg/mL)
1	2.1	341 [M − H]^−^ 387 [M + FA−H]^−^ 179 [caffeic − H]^−^ 683 [2M − H]^−^	365 [M + Na]^+^	342	C_15_H_18_O_9_	Caffeic acid hexoside [39]	14.47 ± 0.44
2	16.6	325 [M − H]^−^ 651 [2M − H]^−^ 163 [*p*-coumaric − H]^−^	349 [M + Na]^+^ 365 [M + K]^+^ 691 [2M + K]^+^ 183 [M + H + K]^2+^	326	C_15_H_18_O_8_	Coumaric acid hexoside [39]	nq
3	17.8	305 [M − H]^−^	307 [M + H]^+^ 329 [M + Na]^+^ 345 [M + K]^+^	306	C_15_H_14_O_7_	Gallocatechin [29,36]	163.77 ± 0.83
4	27.8	477 [M − H]^−^ 955 [2M − H]^−^ 315 [M – H − 162(hexose)]^−^	479 [M + H]^+^ 501 [M + Na]^+^ 979 [2M + Na]^+^	478	C_22_H_22_O_12_	Isorhamnetin 3-O-hexoside [35,36]	43.47 ± 1.22
5	32.4	461 [M − H]^−^ 923 [2M − H]^−^ 299 [M – H – 162 (hexose)]^−^	463 [M + H]^+^ 485 [M + Na]^+^ 947 [2M + Na]^+^	462	C_22_H_22_O_11_	Homoplantaginin (Hispidulin-7-glucoside) [23,36,37]	nq
6	33.7	359 [M − H]^−^ 719 [2M − H]^−^	383 [M + Na]^+^ 743 [2M + Na]^+^	360	C_18_H_16_O_8_	Rosmarinic acid ^st^	111.75 ± 1.24
7	34.5	461 [M − H]^−^ 923 [2M − H]^−^ 285 [luteolin − H]^−^	463 [M + H]^+^	462	C_21_H_18_O_12_	Luteolin -3-O-acetyl-O- glucuronide [37]	88.57 ± 1.85
8	41.0	503 [M − H]^−^ 1007 [2M − H]^−^ 285 [luteolin − H]^−^ 390 443	505 [M + H]^+^	504	C_23_H_20_O_13_	Luteolin-3-O-(O-acetyl)-β-D-glucuronide isomer I [36,37]	16.27 ± 2.94
9	41.8	623 [M − H]^−^	625 [M + H]^+^ 647 [M + Na]^+^	624	C_28_H_32_O_16_	Isorhamnetin-rutinoside	nq
10	42.3	285 [M − H]^−^ 571 [2M − H]^−^	287 [M + H]^+^	286	C_15_H_10_O_6_	Luteolin [37,38]	5.02 ± 2.23
11	42.6	315 [M − H]^−^	317 [M + H]^+^	316	C_16_H_12_O_7_	Isorhamnetin [29,37]	nq
12	43.0	207 [M − H]^−^	209 [M + H]^+^ 231 [M + Na]^+^	208		Trihydroxy cinnamic acid derivative [29]	35.06 ± 0.06
13	46.7	329 [M − H]^−^	353 [M + Na]^+^ 683 [2M + Na]^+^	330	C_17_H_14_O_7_	Cirsiliol	11.77 ± 1.2
14	49.7	345 [M − H]^−^ 691 [2M − H]^−^	347 [M + H]^+^ 715 [2M + Na]^+^	346	C_20_H_26_O_5_	Rosmanol [23,29,36,37,38]	6.52 ± 0.1
15	51.0	345 [M−H]^−^ 691 [2M − H]^−^	347 [M + H]^+^ 715 [2M + Na]^+^	346	C_20_H_26_O_5_	Rosmanol isomer [23,36,37]	40.25 ± 0.11
16	52.4	345 [M − H]^−^ 691 [2M − H]^−^	369 [M + Na]^+^ 715 [2M + Na]^+^	346	C_20_H_26_O_5_	Epirosmanol [23,36,37]	14.05 ± 0.1
17	52.6	359 [M − H]^−^	393 [M + Na]^+^	360	C_18_H_16_O_8_	Epirosmanol methyl ether [36,37]	nq
18	53.7	343 [M − H]^−^ 389 [M + FA − H]^−^	367 [M + Na]^+^ 711 [2M + Na]^+^	344	C_20_H_24_O_5_	Rosmadial [23,36,38]	15.77 ± 0.12
19	54.2	359 [M − H]^−^	361 [M + H]^+^ 383 [M + Na]^+^	360	C_18_H_16_O_8_	Epirosmanol methyl ether [37]	1.31 ± 0.12
20	55.0	329 [M − H]^−^ 375 [M + FA − H]^−^	331 [M + H]^+^ 353 [M + Na]^+^ 683 [2M + Na]^+^	330	C_20_H_26_O_4_	Carnosol [37,39]	nq
21	55.5	359 [M − H]^−^	361 [M + H]^+^ 383 [M + Na]^+^	360	C_18_H_16_O_8_	Rosmanol methyl ether isomer [37]	9.72 ± 0.13
22	60.7	403 [M + H]^−^	427 [M + Na]^+^ 831 [2M + Na]^+^	404		Unknown	27.97 ± 0.34 ^#^
23	63.7	373 [M − H]^−^	375 [M + H]^+^ 397 [M + Na]^+^	374	C_22_H_30_O_5_	11,12-Dimethyl rosmanol [37]	12.66 ± 3.83
24	75.2	345 [M − H]^−^	369 [M + Na]^+^ 715 [2M + Na]^+^	346	C_20_H_26_O_5_	12-*O*-Methyl carnosic acid [37]	3.1 ± 0.11

Notes: nq: not quantified, ^st^: standard compound used for identification, FA: formic acid. ^#^: this compound, although unknown, was quantified with rosmarinic acid curve. The superscript numbers refer to the previous studies on leaves of *Rosmarinus officinalis* that mention the same ingredient.

**Table 3:** Antioxidant activity of rosemary samples expressed as equivalent standard compounds with different antioxidant capacity assays (DPPH, ABTS, FRAP).

Rosemary	DPPH	ABTS	FRAP
Sample	mg BHT/mL	mg Trolox/mL	mg FeSO_4_ × 7H_2_O/mL
Essential Oil	15.10 ± 4.75	2.21 ± 0.11	22.84 ± 2.32
Extract	1.04 ± 0.06	0.25 ± 0.02	0.52 ± 0.05

Note: BHT: butylhydroxytoluene.

**Table 4:** Cytotoxicity of rosemary essential oil and extract on A549 cells.

Essential Oil Concentration	Effect	Extract Concentration	Effect
100%	Cytotoxic	100%	Non-cytotoxic
5%	Non-cytotoxic	90%	Non-cytotoxic

**Table 5:** Effectiveness of rosemary essential oil and extract against various concentrations of Adenovirus.

Essential Oil	AdV 10^9^ PFU/mL	AdV 10^8^ PFU/mL	AdV 10^7^ PFU/mL	AdV 10^6^ PFU/mL	AdV 10^5^ PFU/mL	Adv 10^4^ PFU/mL
5%	+	+	+	+	+	+
Extract						
90%	+	+	+	+	+	+
50%	+	+	+	+	+	+
*25%*	+	+	+	+	+	+

Note: +: Effect against Adenovirus.

## Data Availability

Not applicable.

## Supplementary Materials

The following are available online at https://www.mdpi.com/article/10.3390/foods10123143/s1, Table S1: Results from toxicity experiments on essential oil and rosemary extracts, Figure S1: GC–MS chromatogram of rosemary essential oil.
