# Supplementary material for: Rosemary Extract and Essential Oil as Drink Ingredients: An Evaluation of Their Chemical Composition, Genotoxicity, Antimicrobial, Antiviral, and Antioxidant Properties
Source: Foods. 2021 Dec 18;10(12):3143. doi: 10.3390/foods10123143 (PMC8700793; doi:10.3390/foods10123143)
Supplement: Supplementary file 1 [file foods-10-03143-s001.zip › foods-1487673-supplementary.pdf]

Table S1: Results from essential oil and rosemary extracts toxicity experiments.

Strain	NEGATIVE CONTROL	Rosemary EO (5%)	Rosemary extract (95%)	POSITIVE CONTROL
TA98	Non toxic	Non toxic	Non toxic	Toxic
TA100	Non toxic	Non toxic	Non toxic	Toxic

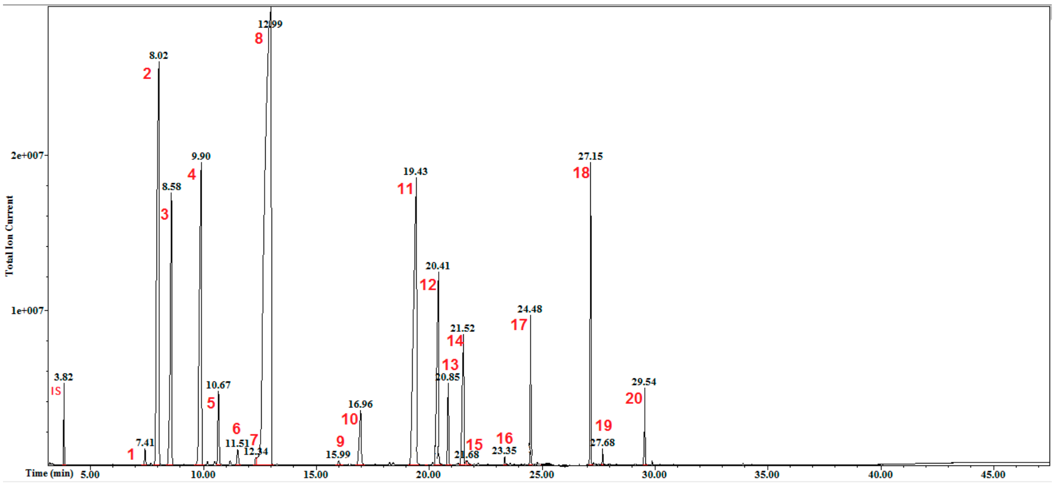

Figure S1: GC-MS chromatogram of rosemary essential oil

IS: Internal Standard (octane), 1: Tricyclene, 2: α-Pinene, 3: Camphene, 4: β-Pinene, 5: Myrcene, 6: 3-Carene, 7: p-Cymene, 8:Eucalyptol (1-8 Cineole), 9: Terpinolene, 10: Linalool, 11: Camphor, 12:Borneol, 13: Terpinen-4-ol, 14: α-Terpineol, 15:Myrtenol, 16:Carvone, 17: Bornyl acetate, 18: E- Caryophellene, 19: α-Humulene, 20: Caryophellene oxide.
